# Personal endotoxin exposure in a panel study of school children with asthma

**DOI:** 10.1186/1476-069X-10-69

**Published:** 2011-08-02

**Authors:** Ralph J Delfino, Norbert Staimer, Thomas Tjoa

**Affiliations:** 1 Department of Epidemiology, School of Medicine, University of California, Irvine (UCI), University of California, Irvine 100 Theory, Suite 100, Irvine, CA, 92617-7555, USA

## Abstract

**Background:**

Endotoxin exposure has been associated with asthma exacerbations and increased asthma prevalence. However, there is little data regarding personal exposure to endotoxin in children at risk, or the relation of personal endotoxin exposure to residential or ambient airborne endotoxin. The relation between personal endotoxin and personal air pollution exposures is also unknown.

**Methods:**

We characterized personal endotoxin exposures in 45 school children with asthma ages 9-18 years using 376 repeated measurements from a PM_2.5 _active personal exposure monitor. We also assayed endotoxin in PM_2.5 _samples collected from ambient regional sites (N = 97 days) and from a subset of 12 indoor and outdoor subject home sites (N = 109 and 111 days, respectively) in Riverside and Whittier, California. Endotoxin was measured using the Limulus Amoebocyte Lysate kinetic chromogenic assay. At the same time, we measured personal, home and ambient exposure to PM_2.5 _mass, elemental carbon (EC), and organic carbon (OC). To assess exposure relations we used both rank correlations and mixed linear regression models, adjusted for personal temperature and relative humidity.

**Results:**

We found small positive correlations of personal endotoxin with personal PM_2.5 _EC and OC, but not personal PM_2.5 _mass or stationary site air pollutant measurements. Outdoor home, indoor home and ambient endotoxin were moderately to strongly correlated with each other. However, in mixed models, personal endotoxin was not associated with indoor home or outdoor home endotoxin, but was associated with ambient endotoxin. Dog and cat ownership were significantly associated with increased personal but not indoor endotoxin.

**Conclusions:**

Daily fixed site measurements of endotoxin in the home environment may not predict daily personal exposure, although a larger sample size may be needed to assess this. This conclusion is relevant to short-term exposures involved in the acute exacerbation of asthma.

## Background

Endotoxin is a cell wall component of the outer membrane of gram negative bacteria. Sources include animals and agricultural activities. In its purified form, it is known as lipopolysaccharide, which is both toxic and immunogenic [1]. A small and variable mass fraction of fine (respirable) particles < 2.5 *μ*m in diameter (PM_2.5_) may contain endotoxin. Experimental inhalation of endotoxin in humans leads to airway inflammation, characterized by activation and migration of neutrophils [2]. Exposure to endotoxin has been associated with exacerbation of respiratory allergic diseases including asthma [1,3], and with increased asthma prevalence [4].

Ryan et al. [5] were the first to show that settled house dust endotoxin and estimated exposure to traffic-related air pollution positively interacted in relation to risk of persistent wheeze at age 3 years in a birth cohort of 624 children. Many other studies have also assessed respiratory and allergic health effects of endotoxin using only house dust samples as a surrogate of subject exposures to airborne endotoxin, often with only one measurement. However, exposure studies have shown considerable within-home, and temporal variability of house dust endotoxin [6-8]. Furthermore, the exposures of interest come from resuspended indoor dust and endotoxin infiltrated from outdoor air that both determine indoor airborne concentrations. Airborne endotoxin measurements are expected to reveal stronger associations between endotoxin and respiratory outcomes than settled dust measurements of endotoxin [9].

Several studies have evaluated household and other determinants of house dust endotoxin [8,10], and of airborne endotoxin inside and outside of the residence of pediatric subjects [11,12]. There is a considerably larger literature regarding airborne endotoxin exposures in occupational settings with organic dusts [13]. However, despite the potential importance of endotoxin in particle-related respiratory health effects, only one study has assessed the impact of personal airborne endotoxin exposure on acute asthma outcomes in children [14]. It is also the only study to have evaluated whether personal endotoxin exposure relates to airborne microenvironmental endotoxin levels among children. Investigators followed a panel of 24 school children with asthma with personal exposure monitors (PEMs) operated at 2 L/min over 24 hours for 164 person-days. They found that personal PM_2.5 _endotoxin and PM_10 _endotoxin exposure was associated with decreased expiratory lung function and increased asthma symptoms. Geometric mean personal endotoxin was higher than indoor or outdoor school levels and was not correlated with these stationary site measurements. This finding suggested that personal endotoxin exposure likely included substantial contributions from other particle sources. Sources include many indoor and outdoor microenvironments and personal dust cloud exposures to particles generated from personal activities (e.g., brushing a dog or yard work) [15] or from exposures to non-stationary sources near the subject (e.g., dust from street traffic).

In the present study we tested the consistency of the personal exposure assessment findings of Rabinovitch et al. [14] using a repeated daily measures in a cohort panel of 45 children with asthma followed over a period of up to 10 days, and using home rather than school endotoxin measurements in a subset of 14 subjects. We also evaluated potential household and other determinants of personal and indoor airborne endotoxin exposures. Data include 376 person-days of daily endotoxin data collected from PM_2.5 _quartz filters using personal exposure monitors operated at 4 L/min, daily ambient endotoxin measurements collected from central ambient sites, and daily indoor and outdoor home endotoxin measurements in a subset of 14 children at 12 residential sites in Riverside and Whittier, California. We also assessed the relationship between personal endotoxin exposures and concurrent personal exposure to air pollutants, including PM_2.5 _mass, PM_2.5 _EC, PM_2.5 _organic carbon (OC), and NO_2_. We then assessed the relationship of personal endotoxin exposures to central site (ambient) measurements of the same air pollutants, and to indoor and outdoor home PM_2.5 _mass, PM_2.5 _EC, and PM_2.5 _OC.

## Methods

### Design and Population

We conducted a longitudinal study with 10 daily repeated measurements of health outcomes and exposures in a panel cohort of school children with diagnosed persistent asthma who were ages 9-18 years (mean 13.5 years), nonsmoking, and unexposed to environmental tobacco smoke in the home. Results relating to asthma outcomes and air pollutants have been previously published [16,17]. Two regional panels were conducted during warmer seasons of southern California. The first panel was conducted in Riverside, California, from August through early October 2003. This is a down-wind smog receptor site, which is a consequence of being just inland from Los Angeles County. The second panel was conducted in Whittier, California from July through November 2004. This is a region of eastern Los Angeles County that is immediately down-wind of vehicular emission sources. Riverside experiences higher temperatures and lower relative humidity than Whittier as a result of being further from the Pacific Ocean and closer to the inland desert.

The Institutional Review Board of the University of California, Irvine approved the study protocol. Informed written consent was obtained from all subjects and one of their legal guardians. Subjects were recruited through notification of parents by local public schools. We recruited only subjects with mild to moderate persistent asthma.

The present study focused on assessing endotoxin exposures in 45 subjects with complete outcome data including four 10-d periods in Riverside involving 13 subjects and eight 10-day periods in Whittier involving 32 subjects. The expected predominance of asthma among males vs. females was evident in this population (14 females and 31 males). This was a diverse population with a majority of subjects identifying themselves as Hispanic (N = 26) along with 5 African American subjects and 14 white non-Hispanic subjects.

### Exposure Assessment

#### Personal Exposure Monitor (PEM)

Subjects carried a PEM during one of the 12 exposure assessment periods of 10 days duration. Three to four different subjects were followed in each of the 12 periods. Subjects were followed-up daily in their homes to download data and exchange PEMs. Each subject wore the PEM during waking hours in a backpack and kept the backpack in close proximity off the ground (e.g. nightstand) when it was not possible to wear it. The air inlets for the PEM were placed over the shoulder strap so that they were close to the breathing zone when worn. The backpack was sound insulated and had an extra compartment for books to be carried during school days. Each day of the 10-day follow-up period, data from an attached motion logger (Onset Computer Corp, Pocasset, MA) was checked to assure compliance. Lack of motion at expected times (e.g. during known school periods), resulted in no monetary compensation to the subject for that day and resulted in the exclusion of data. This occurred on < 6% of person-days of follow-up. Additional filters were not assayed for endotoxin due to air sampler malfunction or problems with filters (10%). Out of 450 expected samples (45 subjects, 10 days per subject), we obtained 376 valid personal endotoxin measurements (83.6%).

Personal measurements included real-time nephelometer mass measurements of PM_2.5 _(personal DataRAM model 1200, MIE Inc., Bedford, MA) and 24-hr average EC and OC fractions of PM_2.5 _collected on quartz filters (Whatman Inc., Florham Park, NJ) using an attached filter cassette. A 2.5 μm sharp-cut cyclone was attached upstream of the nephelometer and PM_2.5 _for EC and OC was collected downstream at a flow rate of 4 L/min. We also measured NO_2 _over 24-hr periods using a miniaturized diaphragm pump run at 0.1 L/min to sample air through triethanolamine-treated molecular sieve sorbent tubes (SKC, Fullerton, CA). We measured NO_2 _based on National Institute for Occupational Safety and Health Method 6014. We also collected personal temperature and relative humidity with attached loggers (Onset Computer Corp, Pocasset, MA). Elsewhere we provide data on the validation of both the personal PM_2.5 _sampler [18], and the personal NO_2 _active sampler [19].

#### Stationary Site Air Monitoring

Harvard Impactors (Air Diagnostics and Engineering, Inc., Naples, ME) were used to collect ambient PM_2.5 _and operated at a flow rate of 10 L/min. They were sited at a central site within 10 km of homes in Riverside and 5 km of homes in Whittier. We also collected indoor and outdoor home PM_2.5 _with Harvard Impactors in one subject's home during each of the 12 ten-day sampling periods. Indoor samplers were located in or near the main activity area of the home, usually the living room or family room. There were a pair of sibling subjects in two of the homes (yielding indoor-outdoor home exposure data for 14 subjects overall for the 12 homes). PM_2.5 _(Teflon filters), and PM_2.5 _EC and OC (Whatman quartz filters) were collected at the stationary sites simultaneous with personal samples. PM mass on Teflon filters was estimated using standard gravimetric methods. For both personal and stationary site quartz filter samples, particulate carbon was speciated into organic and elemental carbon using the thermal manganese dioxide oxidation technique [20]. Criteria pollutant gases were measured by the South Coast Air Quality Management District at central sites and they included hourly O_3 _and NO_2_.

#### Endotoxin Assay

Endotoxin was measured from extracts of archived PM_2.5 _quartz filters (stored at -30°C) collected as described above (376 personal PM_2.5 _filter samples, and 317 central site, indoor and outdoor home PM_2.5 _filter samples). We do not have quartz PM_10 _samples. Although endotoxin is found in the coarse PM fraction (2.5-10 μm in diameter), the respirable PM_2.5 _fraction is more relevant to lower airway dose and thus airway inflammation. All quartz filters were baked to remove organic carbon before sampling. Only around 10% of the filters' surface area was punched out using heat sterilized instruments for the EC-OC measurements, leaving sufficient filter media for endotoxin assays. The remaining surface area for personal endotoxin measurement was calculated for each filter to estimate particle mass using mass data from the 24-hr average PEM PM_2.5 _or gravimetric measurements from the Harvard Impactor PM_2.5 _Teflon filters for the stationary site measurements.

For the endotoxin assay, we developed a rapid and thorough method of extracting endotoxin from quartz PM_2.5 _filters. Briefly, the extraction procedure combines the efficient disruption of quartz filter membranes by using a high speed, reciprocating instrument (FastPrep, MP Biomedicals, Inc., Solon, OH) with conventional sonication. First, the quartz filters were transferred into pyrogen-free extraction tubes with 4 mL pyrogen-free water. The tubes were loaded into the FastPrep and processed at 6.5 m/second for 60 seconds to efficiently homogenize the filter membrane. The extraction tubes were then rotated for 30 min (Dynal Biotech^®^, speed 36) followed by 15 minute sonication and clearing of the aqueous extracts of quartz fibers and particles by centrifugation (at 4000 rpm for 5 min, 4 °C). The undiluted supernatants were then directly used for endotoxin assay using the Limulus Amoebocyte Lysate kinetic chromogenic assay according to the manufacturer's protocol (Pyrochrome Associates of Cape Cod, Falmouth, MA). Negative control quartz filters (field blanks) were extracted and analyzed with each set of air samples. The detection limit for the overall method was estimated at 0.004 endotoxin units (EU)/m^3 ^air (non-detects were set to half this).

### Analyses

Descriptive analyses of exposures were used to determine the shape of the distribution, central tendency, and spatial trends (two regions). We examined the Spearman rank correlation of personal endotoxin to ambient endotoxin measured at a central site in the 45 subjects, and to outdoor and indoor home endotoxin in the subset of 14 subjects. This was intended to establish the extent to which fixed site home and regional measurements are related to personal endotoxin exposure. Similar to other studies [6,8,12] we found notable regional differences in concentrations and in correlations between Riverside and Whittier. Therefore, we present these correlation results separately for the two regions. House dust samples for endotoxin were not collected because the study objective of the parent project was to assess daily acute changes in asthma outcomes and airborne exposures.

Because the endotoxin data for all measurement types were log-normally distributed, we used natural log transformation of the endotoxin variables prior to all regression analyses. We first examined the relation of indoor to outdoor endotoxin in linear regression models. Multiple regression analyses of the relation of continuous log-transformed personal endotoxin to stationary endotoxin measurements were conducted using the general linear mixed model. The mixed model estimates both fixed and random effects [21] and incorporates the basic longitudinal design of the study in which multiple measurements are taken on each subject. Subject random intercepts were modeled to reflect the principle that measurements taken for the same individual are likely to be correlated (not independent).

The following *a priori *adjustments were made in the mixed models for prediction of personal endotoxin by stationary site endotoxin: personal temperature and relative humidity (both significantly and inversely associated with personal endotoxin), and study region. We fit an autoregressive-1 correlation structure given the observed error covariance. Analyses were conducted across both regions (additionally adjusting for region) and separately by region given the difference noted above.

We also used mixed models to analyze the prediction of personal and indoor endotoxin exposure by the following household characteristics: dog and cat ownership, including the number of dogs or cats and whether dogs or cats were allowed in the house (never, occasionally, or often); number of people living in the home, carpet (percentage, age, and regularity of cleaning), whether it was it customary to remove shoes before entering the home, observed cockroaches, observed rodents, flooding damage, surface mold or mildew, livestock, central air-conditioning, and region (Whittier vs. Riverside). Personal and family characteristics were also used in the prediction models and included age group (13-18 vs. 9-12 years since we expected activities and thus exposures to differ between teenagers and younger children), sex, race-ethnicity, mother's education, and family income. For predictor variables in the indoor endotoxin models, we found insufficient variability across the 12 homes for the more refined categories used in the personal models (small cells). Therefore, we dropped carpet cleaning, cockroach and rodent presence, shoe removal, livestock, and air-conditioning. We also dichotomized cat and dog ownership and family income.

We began with crude prediction models adjusted for personal temperature, personal relative humidity and study region for personal endotoxin, and study region for indoor endotoxin (indoor temperature and relative humidity were not associated with indoor endotoxin). We then selected the best multivariate model based on stepwise backward elimination of predictors with the largest p-value over 0.05, and on model fit by AIC. Removed variables were added back singly to the final model to test the appropriateness of the final model.

## Results

### Descriptive analyses of endotoxin and air pollutant exposures

We found detectable endotoxin concentrations in 376 daily personal PM_2.5 _filters analyzed [median 0.57, range 0.002 - 25.3 EU/m^3^]. All 52 personal field blank filters showed low or non-detectable endotoxin (median 0.004 EU/filter). Within-subject coefficients of variation for personal endotoxin ranged from 69% to 224% (median 116%).

We also successfully extracted and found detectable endotoxin concentrations in all 317 daily Harvard Impactor filters from the stationary site active samplers. As described in Table 1, these included 97 ambient, 109 indoor and 101 outdoor home filters, and 10 filters from a site in Whittier that served as both an outdoor home and ambient site during one 10-day run, and served as the central ambient site for remaining 10-day runs. The 42 blank filters at the stationary sites showed low or non-detectable endotoxin (median 0.011 EU/filter). For the comparisons with available indoor and outdoor measurements there were 116 and 113 personal endotoxin measurements, respectively, among the 14 subjects living in those 12 homes. For the comparisons with available ambient endotoxin measurements there were 339 personal endotoxin measurements among the 45 subjects. For the analysis of personal vs. fixed site endotoxin in regression models, one subject for just one day lacked personal temperature and humidity as covariates leaving 338 ambient, 115 indoor, and 112 outdoor observations for analysis. There were all or nearly all 376 personal endotoxin measurements for the comparisons with available ambient air pollution. Ambient air pollutant measurements were nearly complete with at least 407 days for each variable available for comparison with the 423 days of ambient endotoxin measurements.

**Table 1:** Study design and sample size

Subject Sample	Personal Daily Endotoxin Exposure	Indoor and Outdoor Home Daily Endotoxin Exposure	Ambient Central Site Daily Endotoxin Exposure
45 subjects	collected 376 subject samples		97 daily samples available on 339 person-days with personal endotoxin

Subset: 14 subjects in 12 homes^1^	116 subject samples included in above count	Collected up to 10 days per subject home, total = 109 indoor and 111 outdoor daily samples.	109 person-days with personal endotoxin included in above count

^**1**^One subject or two sibling subjects were selected during each of sixteen 10-day sessions with a group of four subjects carrying personal exposure monitors. Thus, indoor endotoxin was available on 116 person-days with personal endotoxin, and outdoor endotoxin was available on 113 person-days with personal endotoxin.

Descriptive statistics regarding all of the exposures by region are shown in Table 2. Arithmetic mean and median personal endotoxin exposures were higher in Riverside than in Whittier. Consistent with this, outdoor home and ambient endotoxin were higher in Riverside than in Whittier. However, indoor endotoxin exposures were higher in Whittier than in Riverside. Although arithmetic mean personal endotoxin was higher than indoor, outdoor or ambient levels across both regions, the median personal endotoxin was only higher in Riverside. This is a reflection of the typical skewed distribution of endotoxin exposures.

**Table 2:** Descriptive statistics of endotoxin and air pollutant exposures by region

	Riverside					Whittier				
**Exposure**	**N^1^**	**Mean (SD)**	**Median**	**IQR.**	**Min/Max**	**N^1^**	**Mean (SD)**	**Median**	**IQR.**	**Min/Max**

**Endotoxin **(EU/m^3^)										

Personal	94	2.30 (3.88)	1.01	2.25	0.003/20.6	282	1.92 (3.67)	0.48	1.97	0.002/25.3

Indoor	31	0.58 (0.42)	0.43	0.68	0.063/1.72	78	1.49 (1.29)	1.10	0.86	0.13/7.5

Outdoor	35	1.46 (1.67)	0.78	1.5	0.12/7.90	76	0.85 (1.18)	0.55	0.89	0.11/9.5

Ambient	34	1.26 (1.17)	0.73	0.88	0.30/4.56	63	0.55 (0.45)	0.45	0.46	0.048/2.51

**Personal Air Pollution and Weather**										

PM_2.5 _(μg/m^3^)	103	30.9 (20.1)	24.7	26.8	6.6/98.4	299	36.4 (26.8)	29.1	21.9	7.6/220.0

EC (μg/m^3^)	132	0.43 (0.61)	0.39	0.32	0.04/6.94	295	0.76 (1.32)	0.47	0.82	0.001/17.2

OC (μg/m^3^)	132	5.95 (2.62)	5.63	3.48	1.94/16.1	301	6.83 (3.41)	6.43	4.05	2.18/31.5

NO_2 _(ppb)	147	23.3 (9.3)	23.4	12.7	5.16/47.6	313	30.6 (14.4)	28.4	18.9	2.7/105.7

Temperature (°C)	147	26.8 (2)	27.2	3.1	22.8/32.1	313	24.8 (2.7)	25.4	3.5	17.3/30.5

Relative Humidity (%)	147	43.9 (8.7)	40.6	14.8	28.6/64.0	312	49.8 (7.0)	50.4	7.75	25.2/66.6

**Indoor Air Pollution and Weather**										

PM_2.5 _(μg/m^3^)	38	14.9 (8.4)	8.5	14.6	5.1/33.8	77	17.4 (10.6)	16.5	8.35	3.59/83.2

EC (μg/m^3^)	37	0.71 (0.30)	0.56	0.41	0.17/1.3	79	0.80 (0.92)	0.62	0.54	0.14/7.75

OC (μg/m^3^)	37	6.2 (1.82)	5.46	2.74	3.17/11.6	79	5.95 (2.37)	5.30	2.27	2.64/13.5

Temperature (°C)	40	28.7 (1.7)	29.3	2.2	24.6/31.7	75	25.6 (2.87)	25.8	3.52	19.8/33.8

Relative Humidity (%)	40	30.2 (10.3)	24.6	13.9	16.7/50.6	75	49.2 (7.21)	51.0	8.16	32.3/62.1

**Outdoor Air Polution**										

PM_2.5 _(μg/m^3^)	38	27.0 (18.6)	15.6	19.4	9.3/71.8	77	19.3 (13.5)	16.4	8.46	3.18/84.3

EC (μg/m^3^)	38	1.10 (0.36)	1.07	0.43	0.50/2.08	79	0.99 (1.41)	0.71	0.62	0.21/12.5

OC (μg/m^3^)	38	6.21 (1.26)	6.07	1.57	3.78/9.66	79	4.54 (1.89)	4.24	2.63	2.05/10.3

**Ambient Air Pollution and Weather**										

PM_2.5 _(μg/m^3^)	40	31.5 (22.1)	17.4	30.1	9.5/87.2	76	17.8 (12.0)	16.0	8.85	2.77/77.1

EC (μg/m^3^)	35	1.55 (0.71)	1.30	0.86	0.52/3.64	76	0.69 (0.44)	0.59	0.45	0.14/2.95

OC (μg/m^3^)	35	6.7 (1.69)	6.07	1.91	4.11/11.6	76	3.89 (1.49)	3.73	2.07	1.64/8.8

NO_2 _(ppb)	40	26.8 (9.9)	26.2	12.3	11.6/54.8	79	27.7 (10.8)	26.0	11.6	12.0/74.1

O_3 _(ppb)	40	77.9 (19.7)	74.5	25.9	33.4/120.8	79	40.7 (14.1)	38.9	18.5	11.1/79.2

Temperature (°C)	40	24.4 (3.6)	25.4	6.05	17.3/30.1	79	20.7 (3.59)	21.2	5.5	11.7/27.8

Relative Humidity (%)	40	27.4 (16.2)	23.0	29	2.0/61.0	79	39.7 (9.64)	42.0	12.0	6.0/61.0

^**1**^This is a repeated measures study so the N refers to the number of exposure measurements, not subjects.

Indoor to outdoor endotoxin ratios of medians were clearly opposite between the two sites with a ratio < 1.0 at Riverside (0.55) and a ratio > 1 at Whittier (2.00). Actual indoor concentrations reflected this difference with a much lower indoor concentration in Riverside than in Whittier.

We show correlation matrixes separately for Riverside and Whittier relating personal endotoxin and stationary site endotoxin (indoor, outdoor and ambient) to personal and stationary site endotoxin and air pollutants (Table 3). We found personal endotoxin in both Riverside and Whittier was not significantly correlated with indoor endotoxin or any of the indoor air pollutants. Personal endotoxin was not significantly correlated with outdoor home endotoxin in either Riverside or Whittier. We observed small positive correlations between personal and ambient endotoxin in Riverside but not Whittier. Outdoor home and ambient endotoxin measurements were strongly correlated.

**Table 3:** Spearman rank correlation matrix of personal and stationary site endotoxin in relation to indoor-outdoor home and ambient endotoxin and air pollutions

		Riverside				Whittier		
	**Personal Endotoxin **	**Indoor Endotoxin**	**Outdoor Endotoxin**	**Ambient Endotoxin**	**Personal Endotoxin **	**Indoor Endotoxin**	**Outdoor Endotoxin**	**Ambient Endotoxin**

Indoor Endotoxin	0.10	1.00	0.46*	0.41*	-0.14	1.00	0.55**	0.52**

Outdoor Endotoxin	0.21	0.46*	1.00	0.83**	-0.19	0.55**	1.00	0.73**

Ambient Endotoxin	0.32**	0.41*	0.83**	1.00	-0.02	0.52**	0.73**	1.00

Personal exposures								

PM_2.5_	-0.24*	0.49*	-0.61**	-0.57**	-0.16**	0.16	0.24*	0.03

EC	0.15	-0.22	0.23	0.31	0.40**	-0.13	-0.08	-0.22*

OC	0.27**	-0.19	0.12	0.05	0.41**	0.03	0.05	0.004

NO_2_	0.13	0.57	-0.07	-0.12	-0.05	0.15	0.21*	0.02

Temperature	0.22*	-0.40	-0.22	-0.27	-0.41**	0.15	0.42**	0.40**

Relative Humidity	-0.04	0.48	-0.22	-0.17	0.09	-0.04	-0.34**	-0.46**

Indoor exposures								

PM_2.5_	-0.14	0.66**	-0.13	-0.19	-0.16	0.27**	0.32**	0.04

EC	-0.18	0.73**	0.02	0.02	-0.10	0.19	0.36**	0.09

OC	-0.20	0.39*	-0.25	-0.36*	0.11	0.30**	0.28**	0.01

Temperature	-0.34	0.11	0.19	0.05	-0.33**	0.04	0.27**	0.29*

Relative Humidity	0.35	0.41*	-0.21	-0.10	0.023	0.11	-0.23*	-0.37*

Outdoor exposures								

PM_2.5_	-0.13	0.80**	-0.03	0.13	-0.26*	-0.12	0.23*	0.05

EC	-0.04	0.24	0.35*	0.52**	-0.02	0.02	0.33**	0.19

OC	-0.37	0.21	0.09	0.23	-0.07	0.02	0.33**	0.14

Ambient exposures								

PM_2.5_	-0.26*	0.70**	-0.15	-0.03	-0.24**	-0.14	0.10	0.03

EC	-0.16	0.03	0.28	0.13	-0.0003	-0.10	0.37**	0.32**

OC	-0.29**	0.14	-0.04	-0.08	-0.15*	-0.07	0.34**	0.31**

NO_2_	-0.25*	-0.01	0.06	0.02	0.04	0.04	0.32**	0.20

Temperature	0.02	-0.64**	0.19	0.18	-0.43**	0.17	0.35**	0.36**

Relative Humidity	0.07	0.54**	0.01	0.12	0.02	-0.12	-0.33**	-0.27**

EC: elemental carbon; OC: organic carbon. * *p  *< 0.05; ** *p  *< 0.01.

In both Riverside and Whittier, personal endotoxin showed a small inverse correlations with personal PM_2.5_, and small positive correlations with personal PM_2.5 _EC and OC, which were larger in Whittier. Personal endotoxin positively correlated with personal temperature in Riverside but negatively correlated with personal temperature in Whittier.

Personal endotoxin in both Riverside and Whittier were not significantly correlated with any of the indoor air pollutants. Indoor endotoxin in Riverside, on the other hand, was strongly positively correlated with indoor PM_2.5 _EC and moderately correlated with indoor PM_2.5 _mass and OC, whereas in Whittier these correlations were positive but much smaller.

Both personal and outdoor home endotoxin in Riverside were not significantly correlated with any outdoor home air pollutant measurement. We observed a small inverse correlation between personal endotoxin and outdoor home PM_2.5 _in Whittier. Outdoor home endotoxin showed small positive correlations with outdoor home PM_2.5_, EC and OC in Whittier.

In Whittier, ambient temperature and O_3 _were negatively correlated with personal endotoxin. In Whittier, but not Riverside, ambient endotoxin showed small positive correlations with ambient traffic-related air pollutants (EC, OC, NO_2_) and temperature and small inverse correlations with relative humidity.

### Regression analyses of endotoxin exposures

The prediction of personal endotoxin in mixed regression models by the various stationary site measurements of endotoxin are shown in Table 4 including both sites together and separately by region. Ambient endotoxin for the 14 subjects in monitored homes, and their exposure to indoor and outdoor home endotoxin were not significant predictors of personal endotoxin. However, ambient endotoxin for all 45 subjects was a significant positive predictor of personal endotoxin. The regional models show that the overall association was attributable to measurements at both sites, although the regression coefficient for Riverside was twice as large as Whittier. However, the regression coefficient for Whittier was more significant than Riverside (*p  *< 0.05 vs. *p  *< 0.1, respectively).

**Table 4:** Associations of personal log endotoxin with indoor, outdoor and ambient log endotoxin^1^

Predictor Variable	N	All Subjects coefficient (95% CI)	N	Riverside coefficient (95% CI)	N	Whittier coefficient (95% CI)
14 Subjects in Indoor-outdoor Monitored Homes,						

Log Indoor Endotoxin	116	0.09 (-0.51, 0.68)	23	0.31 (-1.09, 1.72)	92	-0.03 (-0.73, 0.67)

Log Outdoor Endotoxin	113	0.17 (-0.32, 0.66)	23	0.32 (-1.12, 1.75)	89	0.15 (-0.41, 0.70)

Log Ambient Endotoxin	109	0.39 (-0.20, 0.98)	23	1.09 (-0.76, 2.94)	72	0.39 (-0.26, 1.04)

All 45 Subjects						

Log Ambient Endotoxin	339	0.37 (0.04, 0.71)**	82	0.66 (-0.07, 1.38)*	256	0.39 (0.02, 0.76)**

* *p  *< 0.1; ** *p  *< 0.05

^**1**^Results of linear mixed effects models show the change in log personal endotoxin for a one log-unit change in the predictor variable, adjusted for personal temperature, personal relative humidity, and region (all-subject model only). The sample sizes (N) represent person-days of observation with non-missing personal temperature and relative humidity.

**Figure 1:**
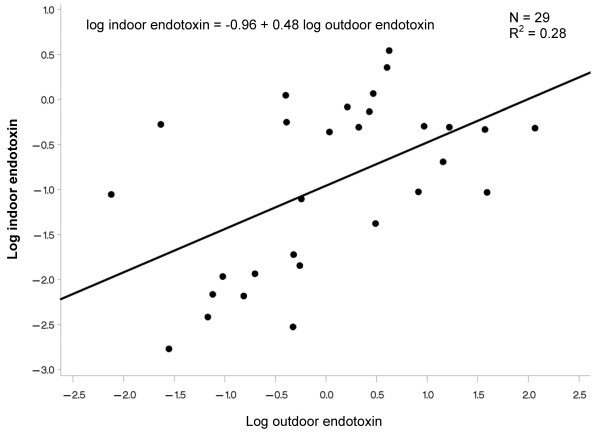
**Relation between indoor and outdoor home endotoxin for 10-day monitoring sessions in 4 homes in Riverside**.

**Figure 2:**
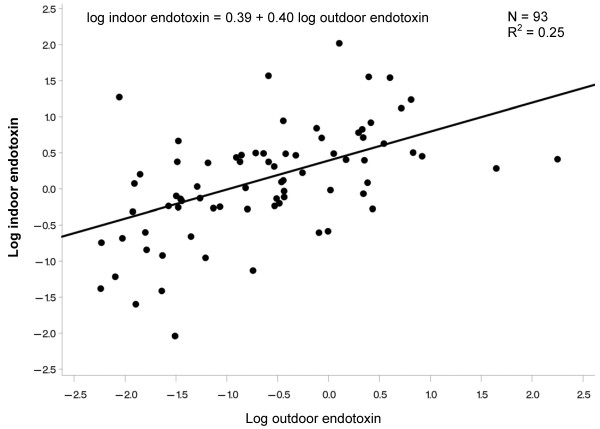
**Relation between indoor and outdoor home endotoxin for 10-day monitoring sessions in 8 homes in Whittier**.

Figures 1-2 show scatter plots and results of linear regression models for the relation between log transformed indoor and outdoor home endotoxin across the 10-day monitoring sessions in 4 homes in Riverside and 8 homes in Whittier. In both regions, the relation was positive, with outdoor endotoxin explaining 25-28% of the variability (R^2^) in indoor endotoxin.

The analysis of the relation between personal endotoxin and household or subject characteristics shows a clear positive association with dog ownership in crude models adjusted for personal temperature, personal relative humidity and region (Table 5). For each dog owned, personal endotoxin exposure approximately doubles. Interestingly, compared with having no dogs, the strongest and only significant association with personal endotoxin in crude models was for dogs that were only occasionally indoors. This contrasts the finding for cats since the only significant association was for having cats that were often indoors compared with having no cats. Other variables were significantly positively associated with personal endotoxin in the crude models, and they included reports of flooding damage (over four times higher personal endotoxin) and sex (males had half the personal endotoxin exposure of females). Nominal associations (p <0.1) included increasing personal endotoxin by the number of household residents and lower personal endotoxin among Hispanics. The final selected multivariate model included only cat and dog numbers adjusted for personal temperature, personal relative humidity and region. We found a relative increase in endotoxin for each dog of 1.76 (95% CI: 1.30, 2.40), and for each cat of 1.39 (95% CI: 1.06, 1.83). Residence of dogs and cats were not included due to expected dependent relations with the number of animals Chi-Square *p*-value < 0.0001). Adding back single excluded variables to this final model did not improve the fit of the model and showed that each of those variables were nonsignificant including flooding damage and male sex (regression coefficients for proportional changes in personal endotoxin were 1.64, *p  *< 0.31, and 0.62, *p  *< 0.2, respectively). Flood and cat were positively associated with each other (Chi-Square *p*-value < 0.02). As a result, cat number confounded the association with flood (decreased by 24.1% from the crude model, p < 0.31) and the association with cats also decreased by 37.5% as well (p < 0.07).

**Table 5:** Proportional change in personal endotoxin exposures related to predictors (household and subject characteristics) in 45 children with asthma

Household and subject characteristics	N or Mean (min-max)	Adjusted coefficient (95% CI)^1,2^
Number of dogs	1.31 (0 - 4)	1.96 (1.43, 2.68)#

Residence of dogs		

No dogs	14	1.00 (referent)

Often Indoors	8	1.20 (0.38, 3.78)

Occas. Indoors	12	4.30 (1.50, 12.27)#

Outdoor Only	11	2.41 (0.77, 7.55)

Number of cats	0.69 (0 - 5)	1.61 (1.19, 2.18)#

Residence of cats		

No cats	31	1.00 (referent)

Often Indoors	10	2.99 (1.14, 7.89)**

Occas. Indoors	2	3.56 (0.53, 23.79)

Outdoor Only	2	2.51 (0.36, 17.57)

Number of residents	4.75 (2 - 7)	1.27 (0.97, 1.65)*

Carpeting		

< 50%	19	1.00 (referent)

≥ 50%	26	0.55 (0.22, 1.37)

Age of carpeting		

< median years	21	1.00 (referent)

≥ median years	18	1.95 (0.78, 4.86)

Frequency of carpet cleaning		

Never/don't know	11	1.00 (referent)

Every 3 years or more	3	0.31 (0.05, 1.90)

More than every 3 years	28	0.89 (0.31, 2.53)

Remove shoes in house		

No	41	1.00 (referent)

Yes, frequent exceptions	2	0.56 (0.08, 4.15)

Yes, few or no exceptions	2	0.61 (0.08, 4.70)

Cockroaches in the home		

No	29	1.00 (referent)

Yes	16	1.54 (0.66, 3.58)

Rodents or droppings		

No	39	1.00 (referent)

Yes	6	0.48 (0.15, 1.55)

Flooding damage		

No	28	1.00 (referent)

Yes	17	4.48 (1.96, 10.26)#

Mold/mildew on surfaces		

No	12	1.00 (referent)

Yes	33	1.34 (0.51, 3.54)

Livestock		

No	43	1.00 (referent)

Yes	2	0.45 (0.06, 3.44)

Central air-conditioning		

No	7	1.00 (referent)

Yes	38	0.55 (0.17, 1.81)

Sex		

Female	15	1.00 (referent)

Male	30	0.43 (0.19, 0.99)**

Age		

13-18 years	31	1.00 (referent)

9-12 years	14	1.18 (0.48, 2.90)

Race		

White non-Hispanic	14	1.00 (referent)

Hispanic	23	0.39 (0.15, 1.05)*

Black	8	0.77 (0.22, 2.68)

Mothers education		

More than high school	31	1.00 (referent)

High school or less	14	1.23 (0.51, 2.97)

Family income		

>50,000	21	1.00 (referent)

30,000-50,000	11	1.96 (0.63, 6.11)

Up to $30,000	13	0.61 (0.24, 1.58)

* *p  *< 0.10, ** *p  *< 0.05, ^# ^*p  *< 0.01

^1^Because the dependent variable (personal or indoor endotoxin) was log transformed, we exponentiated the regression coefficient of the predictor, thus yielding the proportional change in endotoxin exposure and 95% confidence interval (CI).

^2^Adjusted for personal temperature, personal relative humidity and study region.

The analysis of the relation between indoor endotoxin and household or subject characteristics shows that unlike the personal exposure models, dog and cat ownership was not associated with indoor endotoxin (Table 6). Only three variables were significant in the crude models, reports of flood damage, which was unexpectedly associated with lower endotoxin, Hispanic subjects associated with higher endotoxin (in contrast to personal endotoxin), and high school or lower education level in mothers that was associated with lower endotoxin. The final selected multivariate model included only flooding damage and lower education levels in mothers.

**Table 6:** Proportional change in indoor endotoxin exposures related to predictors (household and subject characteristics) in 12 homes of children with asthma

Household and subject characteristics	N or Mean (min-max)	Adjusted coefficient (95% CI)^1,2^
Dog Ownership		

No dogs	4	1.00 (referent)

One or more dogs	8	0.92 (0.37, 2.31)

Cat Ownership		

No cats	9	1.00 (referent)

One or more cats	3	0.65 (0.26, 1.60)

Number of residents	4.42 (2 - 6)	1.15 (0.74, 1.76)

Carpeting		

< 50%	5	1.00 (referent)

≥ 50%	7	0.64 (0.31, 1.33)

Age of carpeting		

< median years	6	1.00 (referent)

≥ median years	6	1.18 (0.55, 2.50)

Flooding damage		

No	8	1.00 (referent)

Yes	4	0.40 (0.24, 0.69)#

Mold/mildew on surfaces		

No	2	1.00 (referent)

Yes	10	0.63 (0.24, 1.66)

Gender		

Female	5	1.00 (referent)

Male	7	1.33 (0.62, 2.87)

Age		

13-18 years	6	1.00 (referent)

9-12 years	6	1.36 (0.61, 3.05)

Race		

White non-Hispanic	3	1.00 (referent)

Hispanic	9	2.35 (1.06, 5.22)**

Mothers education		

More than high school	9	1.00 (referent)

High school or less	3	0.39 (0.21, 0.71)#

Family income		

>50,000	8	1.00 (referent)

Up to $50,000	4	1.24 (0.50, 3.09)

Temperature (°F)	80.0 (67.6 - 92.8)	1.02 (0.98, 1.08)

RH (%)	42.6 (16.7 - 62.1)	1.00 (0.97, 1.03)

* *p  *< 0.10, ** *p  *< 0.05, ^# ^*p  *< 0.01

^1^Because the dependent variable (indoor endotoxin) was log transformed, we exponentiated the regression coefficient of the predictor, thus yielding the proportional change in endotoxin exposure and 95% confidence interval (CI) and 95% confidence interval (CI).

^2^Adjusted for study region.

## Discussion

Our results suggest that fixed site measurements of endotoxin in the home environment may not adequately represent daily personal exposures. The finding of a positive association between ambient and personal endotoxin exposure (45 subjects) is not particularly relevant to research used to investigate relations of respiratory health to endotoxin (usually from indoor sources and activities), but it does have some relevance regarding potential impacts of regional sources on personal exposure. It is possible that the limited sample size (14 subjects, with 112-115 indoor-outdoor home measurements) was insufficient to detect an association of personal with home endotoxin. Evidence in support of that view is that when we limited the analysis of prediction of personal endotoxin by ambient endotoxin to the monitored homes (14 subjects), associations were nonsignificant but point estimates were similar to those for the 45 subjects (Table 4). Nevertheless, although we had a limited sample size in the 14 subjects, the findings for the relation of personal endotoxin exposure with indoor home endotoxin exposure (often the location of sampling in health studies), suggest that other microenvironments and personal activities are important to assess. Given that our analysis was based on daily exposures using measurements all conducted with active 24-hour samplers, our conclusion that any one fixed site measurement may not adequately represent personal exposure applies to short-term exposures that may be involved in the acute exacerbation of asthma. We assessed the potential importance of other locations and physical activity by using previously reported data on quarter-hourly time-activity reports from an electronic diary that each subject filled out throughout follow-up [22]. We found that on average, around 73% of time was spent at home indoor, 1.7% at home outdoor, 12.6% at school indoor, 1.8% at school outdoor, 4% in-transit, 4.3% indoor elsewhere, and 2.6% outdoor elsewhere. Out of an estimated average of 40 min per day of diary-reported moderate to strenuous activity (validated with actigraph data [22]), 82% occurred while away from home. Such higher levels of activity may be important in promoting personal endotoxin exposure as a result of the so-called "personal dust cloud." This is a phenomenon where localized personal activities lead to increased PM exposure by re-suspension of settled PM, which brings the breathing zone of subjects into closer contact with PM from various sources. The highly skewed distribution of personal endotoxin we observed may be partly due to the generation of personal clouds that results from subject activity, including activity around sources of resuspended dust.

Our findings of a general lack of correlation between personal and home microenvironmental endotoxin are consistent with the findings of Rabinovitch et al. [14]. In a panel of school children with asthma, they found geometric mean personal endotoxin was higher than indoor or outdoor school endotoxin levels, and personal endotoxin was not correlated with these stationary site measurements.

Dogs have been identified as a major identifiable source of endotoxin [1]. The present results show a positive association between personal endotoxin and the number of dogs and cats owned, as expected, and this substantiates the utility of the personal exposure measurements. This finding is consistent with a substudy of 10 children by Rabinovitch et al. [23] who found personal endotoxin exposures were significantly higher in 3 households with dogs and one with cats compared with 6 households with no furry pets. We found the association of personal endotoxin was strongest among subjects with dogs that were only occasionally indoors. This could be attributed to entrainment of debris from the outdoor environment into the indoor environment, including fecal matter. However, we found no association between indoor endotoxin and dog or cat ownership. This may be due to either the smaller sample size (12 homes vs. 45 subjects) or that personal exposure is more dynamic as would be expected from the generation of personal dust clouds. Few other household or subject characteristics were significant predictors of personal endotoxin exposure in crude models (flooding damage and sex) and all were confounded by dog and cat ownership. Significant predictors of indoor endotoxin in final multivariate models only included flooding damage and lower education levels in mothers that were unexpectedly associated with lower endotoxin.

Overall evidence, including a lack of prediction of personal endotoxin by indoor-outdoor home endotoxin, and the association between personal endotoxin and dog and cat ownership, supports the view that personal dust cloud exposures may be the predominant driver of personal endotoxin exposure. A study supporting this view monitored rooms of 20 northern California homes and showed that indoor concentrations of both particles and endotoxin in PM_2.5_, but especially PM_2.5-10 _and PM >10 *μ*m, were significantly elevated during the day and were higher with greater levels of subject activity [24]. The study of 10 children by Rabinovitch et al. [23] also found that in children not owning pets, personal endotoxin exposure was nominally higher on days that they reported playing with furry animals (median 0.07 vs. 0.04 EU/m^3^, *p *= 0.08).

We also conclude that personal endotoxin exposure can vary between regions (Riverside had higher concentrations than Whittier). The regions are characterized by large differences in southern California weather, with Riverside being a hot inland area and Whittier being a milder climate with greater influence from the Pacific Ocean. Regional differences in correlations of personal endotoxin with both personal and ambient temperature, as well as regional differences in indoor/outdoor endotoxin ratios may have resulted from this regional difference in weather and major local sources (see below). However, we cannot rule out unmeasured differences in the homes of subjects between these two regions or differences in other microenvironments of the subjects not evaluated such as schools. We did observe a positive linear relation between outdoor and indoor home endotoxin that was small but significant and similar between the two regions (Figures 1-2). Regional differences in airborne endotoxin concentrations across indoor and outdoor sites were also found in a comprehensive study in Fresno, California, which is a city located in the San Joaquin Valley farming region [12]. Authors found spatial variation in endotoxin was moderately explained by proximity to cropland, pasture land, and confined animal-feeding operations. It is notable in this regard that Riverside, which had higher personal, outdoor home, and ambient endotoxin concentrations than Whittier, is nearer to farmland and confined animal-feeding operations.

We observed small positive correlations of personal endotoxin with traffic-related air pollutants (PM_2.5 _EC and OC) especially in Whittier, which has a greater impact of local traffic. Total personal and ambient PM_2.5 _mass showed small inverse correlations with personal endotoxin. No significant correlation between personal PM_2.5 _mass and personal endotoxin was found in a study of 10 children by Rabinovitch et al. [23], which was a substudy of the epidemiologic investigation discussed above [14]. In Whittier, but not Riverside, ambient endotoxin also showed small positive correlations with ambient traffic-related air pollutants (EC, OC, and NO_2_), but negative correlations with ambient temperature and ozone. These observations for both personal and ambient data might be attributable to re-suspension of fine and coarse dust laden with bioaerosols along nearby roadways, which also generate higher concentrations of the traffic-related pollutants, especially during periods of air stagnation and cool temperatures. We previously reported moderate correlations between coarse PM mass and PM_2.5 _black carbon in the study region [25]. This potential source of endotoxin could lead to high spatial variability in resuspended dust containing endotoxin between homes and between other locations near vs. far from busy roadways. In the Fresno study by Tager et al. [12], investigators found only the coarse PM mass fraction (2.5-10 *μ*m in diameter, PM_2.5-10_) was correlated with PM_10 _endotoxin. PM_10 _endotoxin was not correlated with PM_2.5 _mass or EC. Similarly, another study of 13 urban and suburban ambient monitoring sites in southern California found that PM_10 _endotoxin was correlated with PM_10 _mass but not PM_2.5 _mass, ozone or NO_2 _[26]. Because we measured endotoxin only in PM_2.5_, we are unable to directly compare our results with either of these two studies.

One limitation is that we did not measure endotoxin in the coarse PM fraction (PM_2.5-10_), which is enriched in endotoxin. Nevertheless, the respirable PM_2.5 _fraction is more relevant to lower airway dose and thus airway inflammation. Another limitation is that the number of indoor and outdoor home samples was limited to 14 of the 45 subjects, and this may have limited the power to assess relations of personal endotoxin to home endotoxin and the relation of indoor endotoxin to various fixed household and subject characteristics. This was not a limitation for the comparison of personal to ambient endotoxin where data from all 45 subjects could be used. Another limitation is that wearing the personal exposure monitor backpack may have altered subjects' activities and potentially affected true personal endotoxin exposure levels. This is likely, for example when playing sports, which makes it impossible to safely carry the backpack. Finally, the standard measurement of endotoxin exposure in studies of chronic asthma is to utilize vacuumed house dust samples for endotoxin testing. We did not assess whether this type of measurement is representative of long-term personal exposure and are unaware of any study that has evaluated this.

## Conclusions

Our results suggest that it may be insufficient to assume that any one fixed site measurement of endotoxin adequately represents personal endotoxin exposure, including measurements in the home environment. This conclusion from the present data only applies to short-term airborne exposures that may be involved in the acute exacerbation of asthma. The association of personal (but not indoor) endotoxin with dog and cat ownership supports the view that personal dust cloud exposures may be the predominant driver of personal endotoxin exposure. We also provide evidence that regional differences influencing ambient endotoxin, including weather and local unmeasured sources, are important to consider in assessing personal endotoxin exposures. The correlation between endotoxin and traffic-related pollution suggest that endotoxin from resuspended fine traffic dust and/or shared meteorological determinants are important determinants of endotoxin exposure in urban areas with dense local traffic.

Given the results of this study, we recommend that personal endotoxin be the exposure measurement of choice for future research on the importance of endotoxin as a risk factor for the acute exacerbation of asthma. Additional research is needed to assess whether home microenvironmental measurements, including vacuumed dust samples, are sufficiently representative of long-term personal endotoxin exposure for the assessment of chronic asthma outcomes, including the development of asthma during childhood. Finally, the information provided in this study will support design development for additional research involving both multi-pollutant and bioaerosol monitoring in cohorts of subjects with asthma to assess the potential health impacts of combined exposures.

## List of Abbreviations

CI: confidence interval; EC: elemental carbon; EU: endotoxin units; OC: organic carbon; PEM: personal exposure monitor; PM: particulate matter; PM_2.5_: fine particulate matter < 2.5 μm in aerodynamic diameter; PM_0.25-2.5_: coarse particulate matter 0.25-2.5 μm in diameter.

## Competing interests

The author declares that they have no competing interests.

## Authors' contributions

RD was the principal investigator, designed and directed the study, and directed the statistical analysis. NS was in charge of field operations, and the laboratory measurements of the airborne exposures. TJ was responsible for data management and carrying out the statistical analysis of the data. All authors contributed to writing the paper and approved the final manuscript.
